# Effectiveness of compassion-based online therapy on suicidal thoughts and pain catastrophizing in female patients with multiple sclerosis in the relapsing–remitting phase

**DOI:** 10.3389/fpsyg.2023.1128308

**Published:** 2023-12-11

**Authors:** Fahimeh Mohamadpour

**Affiliations:** Department of Clinical Psychology, Faculty of Educational Sciences and Psychology, Shiraz University, Shiraz, Iran

**Keywords:** compassion-based therapy, online therapy, suicidal thought, pain catastrophizing, multiple sclerosis

## Abstract

**Introduction:**

According to research, multiple sclerosis is related to suicidal thoughts and pain catastrophizing as psycho-pathological variables, and on the other hand, compassion-based treatment can reduce mental disorders by targeting dimensions such as self-compassion. Also, since this disease is progressive and over time can cause movement restrictions in these people, online psychotherapy can be a better option for these people. So, the purpose of this study was to investigate the effectiveness of online compassion therapy on suicidal thoughts and pain catastrophizing in female patients with multiple sclerosis.

**Methods:**

The current research was applied and quasi-experimental in a pre-test-post-test manner with a control group. The research sample consisted of 30 patients with multiple sclerosis living in Shiraz in 2019, who were randomly divided into two 15-person experimental and control groups. The participants were tested on scales of suicidal thoughts and pain catastrophizing, and the treatment plan based on compassion therapy was presented to the participants of the experimental group in 8 two-hour sessions online. The control group was placed on the waiting list until the completion of the treatment sessions and the post-test implementation, and after the post-test implementation, they received the treatment. Control variables in this study included gender and disease phase. Then, their scores were measured, after completing the treatment, and a three-month follow-up period. Descriptive statistics and covariance test were used to analyze the data.

**Results:**

Patients showed a clear improvement in the severity of symptoms in both variables. So that the scores of suicidal thoughts and pain catastrophizing in the experimental group after receiving the treatment, as well as after a three-month follow-up period, were significantly reduced (*p* < 0.001).

**Conclusion:**

Confirming the effectiveness of online therapy based on compassion on improving psychological outcomes in these patients, as an effective and online treatment method, is a clear step towards continuing the implementation of psychological interventions and paying attention to the dimension of mental health in this group of people.

## Introduction

Multiple sclerosis (MS) is the most common debilitating disease, the prevalence of which is increasing in both developed and developing countries, while its cause is still unclear (Dobson and Giovannoni, 2019). In its progressive and chronic course, MS disease, by damaging the myelin tissue of the brain and spinal cord, leads to a wide range of neurological symptoms (Malygin et al., 2019) and also affects the physical, mental, and social functioning of patients (Kotan et al., 2019). This disease is more common among women. Since the early 1900s, the sex ratio has been steadily increasing and now approaches 3:1 (F:M) in most countries (Dobson and Giovannoni, 2019). Also, the most common age of onset of this disease is 20–40 years, which includes the peak of a person’s social and family responsibilities (Nyland et al., 2019).

There are different types of MS disease, but the symptoms of the disease differ among people (McGinley et al., 2021). In general, this disease can be divided into four main categories: (a) relapsing–remitting type: usually the disease begins with this type. In this type, the attacks are unpredictable and the intensity of the attack can be very mild to very severe. (b) Primary progressive type: patients do not relapse or subside, and from the very beginning of the disease, their disability gradually increases. (c) Secondary progressive type: the patient becomes weaker over time without having clear periods of attack or remission. (d) Progressive-relapsing type: A rare type of MS that begins with a progressive course and relapsing periods are added to the course (Ross et al., 2022).

In multiple sclerosis disease, in addition to physical symptoms, many psychological problems also appear, which both affect the mental health of the person and can lead to the exacerbation of physical symptoms in the course of the disease. Therefore, examining the effectiveness of psychological interventions on these patients, in addition to identifying effective treatments, can also help in creating research-based evidence for the effect of these treatments (Davoodi et al., 2019). The relationship between psychological and psychiatric disorders and MS is complex. They can aggravate the physical symptoms related to this disease, such as physical disability, suffering and dysfunction of family and social life (Holland et al., 2019). According to the conducted researches, the coexistence of psychiatric disorders in this disease is clearly common and most of this coexistence is related to depression and anxiety diseases (Nasiri et al., 2016). Depression is one of the most common risk factors for suicide (Kalb et al., 2019). Studies indicate a high prevalence of suicide among patients with multiple sclerosis, and suicide attempts occur in more than 50% of patients (Brenner et al., 2016). While despite the high prevalence of suicide risk, screening these patients for suicidal thoughts and then performing psychotherapy and psychiatric measures to reduce this risk is often not included in the first line of treatment (Shen et al., 2019).

The suicidal process can be considered as a potential progression from ideation to attempt or action to die (Söderholm et al., 2020). Suicidal behavior includes suicidal thoughts (ideas/thoughts), plans, attempts, and finally death through a specific action (Ji et al., 2020). Although not all suicidal thoughts ultimately lead to suicide attempt or suicide, it is the first step on the path to suicide. Therefore, it is considered one of the strong indicators of suicide in the future (Pandey et al., 2021). Suicidal ideation (SI), often called suicidal thoughts or ideation, is a broad term used to describe a range of thoughts, wishes, and preoccupations with death and suicide (Harmer et al., 2022). According to researches, during acute symptoms of this disease and negative emotions caused by these symptoms, the risk of suicidal thoughts increases (Brown et al., 2020).

Meanwhile, another common symptom in patients with multiple sclerosis is pain, which according to research evidence is experienced by more than 50% of these patients. But scientific evidence shows that these patients also experience high levels of pain catastrophizing along with chronic pain (Huang et al., 2022). Pain catastrophizing is a set of negative and exaggerated cognitive and emotional schemas related to a real or expected painful situation, which, as a cognitive deviation, leads to a negative and irrational prediction of the event in the future (Wilson et al., 2023).

Considering the negative impact of mental disorders on the course and severity of this disease and the need to control it, the interaction between physical treatments related to this disease and its psychological treatments seems essential (Ehde et al., 2019). One of the new therapeutic interventions that can be effective in improving the mental health of patients with MS is compassion-focused therapy. Two basic goals in this treatment include reducing self-directed hostility and developing the individual’s abilities to create a sense of self-assurance, kindness, and self-soothing (Gilbert, 2014). Experiencing a compassionate attitude helps people to feel a sense of commonality between themselves and others and thereby overcome their painful feelings (Andersen and Rasmussen, 2017). Therefore, people in compassion-based therapy can recognize their emotional experience in the first step. Then have a compassionate attitude to their situation, which requires adopting a balanced mental perspective called “mindfulness.” Mindfulness is a balanced state of consciousness that requires full observation and acceptance of the emotional and psychological conditions that arise. When a person is not aware of their painful thoughts, they do not accept their experiences as they are. This refusal can show itself in the form of preventing these thoughts from being brought to consciousness and finally, incompatibility with the existing conditions. In this treatment method, instead of focusing on changing people’s “self-evaluation,” people’s relationship with their self-evaluation is changed (Cleare et al., 2019). So, according to this therapeutic approach, pain (both physical and emotional) is often a part of the human experience that can lead to thoughts of self-harm, self-criticism, and guilt. As a result, the basis of this approach is based on exercises that lead to balancing the experience of emotions as a result of the occurrence of pain (Frostadottir and Dorjee, 2019). SC, on the other hand, is an affiliative affect regulation process that fosters warmth and soothing and deactivates the threat focused affect systems (Lanzaro et al., 2021). Also, It has been hypothesized that compassion may be rooted in affiliative affect regulation systems that control pain modulation processes and neurotransmitters (e.g., oxytocin, vasopressin) (Lanzaro et al., 2021). That these cases can also affect the reduction of pain catastrophizing in patients.

Many studies have shown that higher self-compassion is associated with lower anxiety and depression (Sommers-Spijkerman et al., 2018) as well as lower psychological distress, greater psychological well-being, and higher resilience to stress (Salimi, 2018). Various researches have shown the effectiveness of compassion-based therapy in reducing anxiety and depression. This treatment has also shown its effectiveness on female patients with MS (Foroutan et al., 2020). Compassion therapy is one of the therapeutic methods that has shown effectiveness in increasing the tolerance of disturbance and pain control (Andersson, 2018). Overall, compared to other psychotherapies, CFT is cost-effective in clinical treatment and often produces positive therapeutic outcomes when used to treat patients with concerns of anxiety, shame, self-criticism, self-harm/suicide, or depression (Schuster et al., 2020). In their review, researchers found that higher self-compassion and self-forgiveness are associated with less self-harm and suicidal thoughts (Cleare et al., 2019) and another systematic review study showed the effectiveness of self-compassion on chronic pain and pain catastrophizing (Lanzaro et al., 2021).

From the 80s to the 90s, along with the development of computers and digital technology, digital mental health interventions have also received research attention (Krieger et al., 2016). The main rationale for using virtual therapies has been to address the large gap in mental health-related treatments, including the lack of therapists, long waiting lines, and most importantly, individual obstacles on the part of clients such as physical disabilities (Amichai-Hamburger et al., 2014), as patients with MS are also involved in this condition especially in the conditions of disease progression. The remarkable thing about compassion-based therapy online is that the paradigm and main framework of this therapy is not much different from its face-to-face therapy, and this is due to the nature of the techniques used in this approach, which is mostly implemented in the form of discussion and exchange of ideas. And in some cases where the techniques are practical, it is in the form of paper and explanation, which does not cause problems to implement it online (Krieger et al., 2016).

It is important to note that the cause of death of 15% of MS patients is suicide, and about a quarter of these patients always have suicidal thoughts (Feinstein and Pavisian, 2017). The presence of suicidal thoughts leads to the loss of useful years of life and threatens the mental health of these people, and it also brings many problems to the society and family of these people (Lewis et al., 2017). Also, pain catastrophizing is one of the variables that alone can increase the physical and mental damage of these people, and can also increase the risk of suicide. As mentioned in the beginning of the introduction, usually the onset of MS is relapsing–remitting type and therefore, patients are more of this category at the beginning of diagnosis. Since this disease is chronic, what is important is increasing the adaptation of these people to their disease and reducing their psychological symptoms, from the beginning of the disease diagnosis. In other words, the earlier psychotherapy starts for them, the more easily the severity of symptoms can be controlled; Especially in the discussion of suicide, early prevention can reduce future harm. On the other hand, the majority of these patients are women, and according to research, suicidal thoughts and pain catastrophizing are more common in this group. Also, this disease is progressive and over time can cause movement restrictions in people, and therefore, this group is one of the priorities for using virtual therapy. All of these factors determine the necessity of providing constructive solutions in order to prevent suicide and reduce the catastrophizing of pain in these patients, especially in the early stages of diagnosis and treatment.

On the other hand, this disease is progressive and over time, it can cause movement restrictions in this people. In the field of Internet-based therapy with compassion therapy approach, not many studies have been conducted so far. While in general, the group of physical patients who need psychological care and psychotherapy, such as MS patients, as high-risk groups, are considered to be the priority for receiving the use of virtual facilities and psychotherapy based on the web, and so, more randomized controlled trials should be repeated to rely on these sources (Amichai-Hamburger et al., 2014). So, the aim of this research was to investigate the effectiveness of online compassion therapy on suicidal thoughts and pain catastrophizing in female patients with multiple sclerosis.

### Purpose of study

The hypotheses of this research include the following:

1. Compassion-based online therapy reduces suicidal ideation in female patients with M.S.
2. Online compassion-based therapy reduces pain catastrophizing in female patients with M.S.

## Methods

The current research was applied and experimental research in the pre-test-post-test method with a control group.

### Participants

The statistical population in this study was the patients with multiple sclerosis (the relapsing–remitting type that had been diagnosed for at least 1 year) living in Shiraz in 2019, whose disease was diagnosed by a neurologist. In order to achieve reliable results in experimental designs, the presence of at least 15 people in each group is recommended (Kumar, 2018). Therefore, among the statistical population of the research, 30 people were selected as the sample size and using the available sampling method from the statistical population.

**Figure 1:**
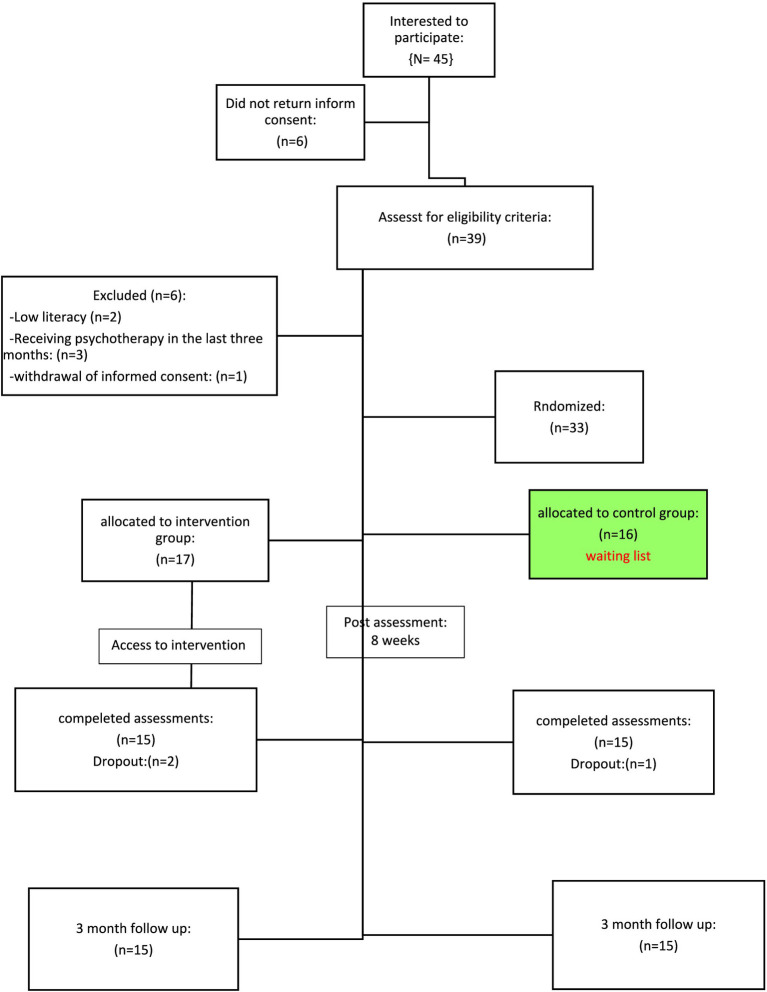
Participant flow.

The criteria for entering the research include female, the relapsing–remitting type, confirmation of the disease by a neurologist, not having a severe and acute stage of the disease, not receiving other psychological treatments during the past 3 months, having at least a diploma level education and having a smartphone in order to participate in the online group. Reluctance to participate in psychotherapy sessions, having psychiatric disorders (including disorders of the psychotic group in DSM-5) based on the diagnosis of a clinical psychologist/psychiatrist and absence of more than two sessions in therapy sessions were also criteria for exiting the study. The flowchart of the participants is presented in Figure 1 at the end of the text.

### Research ethics

In order to comply with research ethics, people were informed about the research objectives, treatment method, the right to withdraw from the treatment process and the confidentiality of information and then completed the informed consent form. Written informed consent was obtained from the individuals for the publication of any potentially identifiable images or data included in this article.

### Procedure

The participants were randomly divided into two groups of 15 people, experimental and control. First, the participants were tested on suicidal ideation and pain catastrophizing scales. Control variables in this study included gender and disease phase. Then, the treatment plan based on compassion therapy was presented to the experimental group participants in 8 two-hour sessions and the control group was placed on the waiting list until the completion of the treatment sessions and the post-test implementation, and after the post-test implementation, they received the treatment. After completing the treatment sessions of both groups, and after a three-month follow-up period, they were tested again and the results were analyzed.

In this research, two techniques have been used to make the research double-blind and, as a result, reduce possible biases. First, a person outside the research team was used as a result assessor to collect the completed scales. Also, the second technique used for double-blinding in this research was the use of random division of people into two experimental and control groups.

### Statistical analysis

Group differences in demographic data were tested with independent samples t-test for continuous variable (age) and chi-square test for nominal variables (marital status, education level and socioeconomic status). To analyze the present results, a linear model was used by predicting the scores after the intervention, controlling the scores before the intervention as a covariate. So, the differences in the results of treatment implementation were evaluated based on one-way analysis of variance (ANCOVA; to compare one dependent variable in two control and experimental groups) and Multivariate analysis of variance (MANCOVA; to compare a scale and its subscales in control and experimental groups), considering treatment conditions as an inter-group factor. This approach includes the use of the covariance structure in the statistical control of hidden confounding variables, using the pre-test effect as the co-variate, and therefore, shows the best information indicators (Meyers et al., 2016).

In order to achieve reliable results in experimental designs, the presence of at least 15 people in each group is recommended (Kumar, 2018). Based on this, the current sample size was considered 15 people for each group. The evaluation of intra-group and inter-group changes due to the implementation of the treatment was compared in the control and experimental groups, and the follow-up evaluation was analyzed only for the intervention group and based on the post scores. Within- and between group effect sizes (Cohen’s d) were calculated based on estimated means and the pooled standard deviation from the observed means. Richards and Richardson (2012) report an averaged effect size of Cohen’s d of 0.78 for guided interventions and of 0.36 for unguided interventions (Richards and Richardson, 2012). Because guidance was used when requested, which could be considered between unguided and fully guided, a medium effect size of 0.50 was aimed. An error level of α 0.05 was also considered as a significance threshold (Ferguson, 1959). All analyses were performed in SPSS version 24.

### Research tools

#### Suicidal ideation scale

Beck’s (1993) standard suicidal ideation scale is a self-report scale that has 19 items on a 0–2 Likert scale. The range of scores of this scale is between 0 and 38, and higher scores indicate a greater risk of suicidal thoughts in a person. According to researches, Cronbach’s alpha coefficient of this scale was 0.97 in the general population and 0.94 in the group at risk (Kliem et al., 2017). Also, the convergent validity of this scale with the Beck depression questionnaire was 0.36 and its divergent validity with the life satisfaction scale was −0.27. This scale has also been measured in Iran, and based on their results, the validity of the scale with the general health questionnaire was 0.76, and its Cronbach’s alpha was 0.95, which indicates its validity and reliability in the Iranian population (Ghaffari et al., 2021).

#### Pain catastrophizing scale

The pain catastrophizing scale was developed by Sullivan et al. (1995) with the aim of evaluating the catastrophic thoughts and behaviors related to pain in people (Sullivan et al., 1995). This scale has 13 items that are set in a self-report manner and includes three subscales of mental rumination, magnification and helplessness. The items of this scale are graded based on a five-point Likert scale from zero (never) to four (always). Lower scores indicate less catastrophizing. The internal reliability of this scale has been reported as 0.94, 0.87, 0.78, and 0.89 for the total score and the subscales of mental rumination, magnification, and helplessness, respectively (Chibnall and Tait, 2005). In Iran, the reliability and validity of this scale has been evaluated and reported as appropriate (Mortazavi-Nasiri et al., 2015).

#### Therapeutic intervention

The researcher has tried to provide a model of intervention in the area of goals, using the therapeutic techniques and exercises recommended in the online compassion-based therapy method presented by Krieger et al. (2016). In each meeting, explanations were first given about the purpose of the meeting and the topics related to that meeting, and then the group members discussed and exchanged opinions. At the end of each session, the materials were summarized and exercises were given to them for the next session. In general, this intervention program was an Internet-based program that included audio files, text, message exchange by members, and also provided exercises related to this therapeutic approach. In Table 1, the protocol of online therapeutic intervention program is presented.

**Table 1:** Treatment program.

Sessions	Objectives	Exercises/techniques
1	Introduction to online CFT How to use the platform and participation in the online CFT Introduction to CFT Familiarization of therapist and group members	Discussions in the online platform Pretest
2	Describing compassion	Teaching mindfulness along with physical examination and inhalation technique
3	Characteristics of compassionate people Being compassionate to others themselves Acceptance and no judgment	Discussion in online platform
4	Self-recognition and recognizing themselves as compassionate or not Assessment of being compassionate to others and themselves	Discussion in online platform
5	Teaching styles of being compassionate to themselves and others	Compassionate talking and behavior Writing a compassionate letter
6	Teaching compassionate skills in the fields of compassionate attention, reasoning, and behavior	Finding self-compassion and self-criticism in inner dialogue and finding similarities in talking with the people most important to themselves
7	Treating fear of self-compassion Assessment of objective and subjective barriers in fostering self-compassion and coping strategies	Self-compassionate mental imaging technique
8	Summary and conclusion Answering possible questions Assessment of all session Posttest	Discussion in online platform Posttest

## Findings

The research sample included 30 female patients with multiple sclerosis. The average age of the participants in this research was 26.59 with a standard deviation of 8.14, and they were randomly assigned to two control and experimental groups. In the current sample, most participants were single (*n* = 20; 33.34%), and some were married (*n* = 10, 66.66%). In terms of years of education, *n* = 23 participants had a bachelor’s degree (76.66%), while *n* = 6 had a master’s degree (20%) and *n* = 1 had a Ph.D.’s degree (3.34%). Also, in terms of socioeconomic status, *n* = 15 participants were average (50%), while *n* = 10 were above average (33.34%) and *n* = 5 were lower than average (16.66%). T-test was used to measure the equality of two groups. The results showed that there is no significant difference between the two groups in terms of average age (*p* = 0.563; *t28* = 0.461). Also, in order to measure the equality of two groups in other demographic variables, Chi-square test was used. In Table 2, the results of the statistical analysis of this test are presented.

**Table 2:** Frequency distribution of demographic information in two groups.

Variable		Experimental: number (%)	Control: number (%)	*χ*^2^ test	*p* value
Marital status	Single	11 (36.66)	9 (30)	0.600	0.439
	Married	4 (13.34)	6 (20)		
Education level	B.A	11 (36.66)	12 (40)	1.710	0.425
	M.A	4 (13.34)	2 (6.66)		
	Ph.D	0	1 (3.34)		
Socioeconomic status	Average	9 (30)	6 (20)	1.200	0.549
	Above Average	4 (13.34)	6 (20)		
	Lower than Average	2 (6.66)	3 (10)		

As the table also shows, there is no significant difference between the two groups in terms of marital status, education level and socioeconomic status as intervening variables.

In Table 3, the descriptive statistics of the research variables are presented separately for the control and experimental groups.

**Table 3:** Mean and standard deviation of research variables in two experimental and control groups.

Research variable	group	Pre-test		Post-test		Follow up	
		Mean	S.D	Mean	S.D	Mean	S.D
Suicidal ideation	Experimental	16.41	4.11	9.73	5.19	8.32	5.12
	Control	16.28	4.32	16.78	5.53	17.39	5.76
Mental rumination	Experimental	18.48	2.81	12.41	3.16	12.89	3.01
	Control	18.21	2.62	18.34	2.98	17.92	3.15
Magnification	Experimental	13.80	2.03	11.72	1.94	10.62	2.25
	Control	14.10	1.98	13.69	2.13	13.53	2.46
Helplessness	Experimental	21.18	1.48	16.56	2.49	15.71	2.34
	Control	21.63	1.88	20.13	2.20	20.89	2.55
Pain catastrophizing	Experimental	53.67	5.59	43.83	5.13	41.91	5.47
	Control	52.93	5.27	52.33	6.29	51.56	6.86

In order to implement the research hypotheses, Kolmogorov–Smirnov test was first performed to check the normality of the data and Leven’s test was also performed to check the equality of variances. Due to the fact that these two tests were not significant in any of the groups, therefore, the assumption of normality of data and equality of variances was conformed for all variables.

In examining the first hypothesis, suicidal thoughts in the post-test were used as dependent variables and group (control, experimental) as independent variables, and suicidal thoughts scores obtained in the pre-test were used as covariate variables. Table 4 shows the effectiveness of compassion-based online therapy on suicidal ideation scores.

**Table 4:** The results of ANCOVA analysis of the mean scores of suicidal thoughts in the experimental and control groups with the pre-test co-variate.

Source of variance	*df*	Mean of squares	*F*	*p*	Effect size
Pre-test	1	368.851	139.376	0.000^**^	0.424
Group	1	42.865	20.249	0.000^**^	
Error	27	1.345	–	–	

^**^p < 0.001.

As shown in Table 4, F obtained from the pre-test analysis is significant (*p* < 0.001). It shows that the pre-test or auxiliary variable is correctly selected and has an effect on the dependent variable. By observing the obtained *F* (20.249) in the groups section, which is related to the difference between the control and experimental groups by removing the effect of the dependent variable, it can be said that the difference between the groups is significant (*p* < 0.001). This suggests that the use of online compassion-based therapy has an effect on suicidal thoughts and the effectiveness of the treatment was 42.4%.

In order to evaluate the effectiveness of treatment on pain catastrophizing and its subscales, multivariate covariance analysis (MANCOVA) was used. Table 5 shows the effectiveness of compassion-based online therapy on pain catastrophizing scores.

**Table 5:** The results of multivariate covariance analysis (MANCOVA) of the difference in mean scores of pain catastrophizing in experimental and control groups with the pre-test co-variate.

Variables	Source of variance	*df*	Mean of squares	*F*	*p*	Effect size
Mental rumination	Pre-test	1	161.201	198.963	0.000^**^	0.573
	Group	1	173.268	231.698	0.000^**^	
	Error	24	135.169	–	–	
Magnification	Pre-test	1	4.150	4.916	0.042^*^	0.193
	Group	1	14.549	5.137	0.032^*^	
	Error	24	14.276	–	–	
Helplessness	Pre-test	1	30.522	8.776	0.005^*^	0.521
	Group	1	32.751	10.385	0.000^**^	
	Error	24	29.412	–	–	
Pain catastrophizing	Pre-test	1	698.169	107.864	0.000^**^	0.589
	Group	1	233.942	34.558	0.000^**^	
	Error	24	6.369	–	–	

^*^p < 0.05; ^**^p < 0.001.

As can be seen in Table 5, the F obtained from the pre-test analysis on the dimensions of pain catastrophizing and its overall score is significant (*p* < 0.05 and *p* < 0.001), which indicates that the pre-test or auxiliary variable is correctly selected and it affects the dependent variable. Also, the obtained *F* (231.698, 5.137, 10.385, 34.558) in the groups section, which is related to the difference between the control group and the experiment by removing the dependent variable, is significant (*p* < 0.05 and *p* < 0.001) which shows the difference between the groups. This shows that the use of compassion-based online therapy has an effect on pain catastrophizing and its dimensions. Also, the effectiveness of the treatment on pain catastrophizing was 58.9% and the effectiveness of the treatment on the variables of mental rumination, magnification, helplessness and pain catastrophizing was 57.3, 19.3, 52.1 and 58.9%, respectively.

Also, in order to check the durability of the treatment effect, the scores of the participants after 3 months from the end of the treatment were measured using the analysis of covariance test (ANCOVA), the results of which can be seen in Table 6.

**Table 6:** The results of the covariance analysis of the difference in mean follow-up scores in the experimental and control groups with pre-test control with the post-test co-variate.

Variables	*df*	Mean of squares	*F*	*p*
Suicidal thoughts	1	372.195	141.653	0.000^**^
Pain catastrophizing	1	269.547	37.167	0.000^**^

^**^p < 0.001.

As seen in Table 6, the effects of online compassion-based therapy on the variables of suicidal thoughts and pain catastrophizing remained after a 3-month follow-up period (*p* < 0.001).

## Discussion

The findings showed that online therapy based on compassion reduces suicidal thoughts and pain catastrophizing in female patients with multiple sclerosis. Few studies have been done on the effectiveness of this type of online treatment. However, these few studies have confirmed the effectiveness of this treatment online in line with the present research (Krieger et al., 2016). Regarding the effectiveness of compassion-based treatment on psychological symptoms in patients with multiple sclerosis, the results of previous studies have indicated the effectiveness of this type of treatment (Salimi, 2018; Frostadottir and Dorjee, 2019; Foroutan et al., 2020; Lanzaro et al., 2021).

In explaining the findings of this research, it can be said that the core of compassion-based therapy is self-compassion, care, understanding, and empathy. For a patient, self-compassion means having a positive attitude toward one’s current situation, especially when there is an uncontrollable condition such as a chronic illness. On the other hand, self-compassion, by cultivating positive emotions in a person, increases hope for the future in a person, and through positive self-evaluation, it is considered an effective protector against inefficient negative emotions such as suicidal thoughts (Gilbert, 2014).

CFT acknowledges that pain (physical or emotional pain) is often part of the human experience, and that the experience of pain can trigger thoughts of self-harm, criticism/condemnation, guilt, or shame. Consequently, CFT aims to modify or transform self-harm/suicide or self-criticism into compassionate self-modification through various tools of compassionate practice, such as self-regulation practices, mindfulness, and compassionate imagery. Also, this therapeutic approach emphasizes that one of the ways to reduce suffering in life is to be kind to yourself and others. Experiencing a sympathetic attitude towards others helps people to feel shared between themselves and others and, as a result, overcome their painful feelings more easily (Andersen and Rasmussen, 2017). Another important dimension in this treatment is awareness and mindfulness, which requires full observation and acceptance of emotional and psychological conditions (Cleare et al., 2019). The reasoning is that when people do not bring their painful thoughts to consciousness, it will lead to incompatibility with the existing situation. On this basis, this approach is based on techniques and exercises that lead to balance in the experience of emotions when pain occurs (Frostadottir and Dorjee, 2019). This balance in the experience of emotions both frees the person from the trap of suicide and reduces the catastrophic nature of the experienced pain, both of which are incompatible emotions with the situation.

Another reasoning for the effectiveness of compassion-based therapy is the physiological effectiveness of this approach and the soothing system that emphasize pain modulation processes and control neurotransmitters (Lanzaro et al., 2021). The result of these physiological effects is to reduce the catastrophic feeling of pain in these people. On the other hand, compassion-based therapy, by enhancing relaxation and deactivating threat-focused systems, can also help reduce catastrophizing pain in these patients. This shows that the effectiveness of this approach, in addition to cognitive and emotional settings, also happens through the physiological path.

What is certain is that due to the limitations of different platforms in the implementation of online therapy, the effectiveness of this type of therapy depends a lot on the therapeutic approach implemented and the techniques used in it. In the online implementation of compassion-based therapy, this type of implementation method follows the main framework of this therapeutic approach and only in some cases, more explanations are needed (Krieger et al., 2016). In addition, the required exercises should be aligned and presented according to the used platform in a way that can be understood and implemented by the clients. Another fundamental difference of this type of therapeutic implementation compared to face-to-face implementation is more and comprehensive emphasis on the principle of confidentiality, especially in the implementation of group therapy. In other cases, it is almost identical to the same treatment points as in-person. Therefore, by observing these points and strictly implementing the instructions, we can expect the same result as online and group treatment, as seen in the present study.

## Conclusion

Compassion-based therapy, due to the nature of the techniques used in it, which is mostly mental and conversation-oriented, can be one of the suitable options for online use and has the necessary ability to be implemented. As the results of the present study also show. Patients with multiple sclerosis, especially in the acute stages of the disease, although they are involved in various psychological conditions and consequences related to their disease, but due to their special physical conditions, they often face problems in face-to-face psychotherapy situations. In addition, considering the current special conditions (the prevalence of the corona pandemic and the need for social distancing) and since these people are at greater risk than others due to the presence of an underlying disease, the review and implementation of online treatments for them, is more important. Therefore, confirming the effectiveness of online treatment based on compassion on improving mental outcomes in these patients, as an effective and non-attendance treatment method, is a clear step towards continuing the implementation of psychological interventions and paying attention to the mental health aspect of this group of people.

### Limitations and future research

While applying the findings of the present study, one of the limitations of this study was the available sampling method, the use of a unisex sample of female patients, and the short-term follow-up period, which makes it difficult to generalize the results. It is noteworthy that the current statistical procedure does not control for the effect of attrition (dropouts), and that future studies should consider following an Intention to Treat (ITT) analysis. Also, it is suggested that in order to develop the findings of the current research, male samples should also be used. Also, in order to evaluate the stability of grades, long-term follow-up periods should also be measured.

## Data availability statement

The original contributions presented in the study are included in the article/supplementary material, further inquiries can be directed to the corresponding author.

## Ethics statement

The studies involving humans were approved by Shiraz University of the ethics committee. The studies were conducted in accordance with the local legislation and institutional requirements. The participants provided their written informed consent to participate in this study.

## Author contributions

The author confirms being the sole contributor of this work and has approved it for publication.
